# The complete mitochondrial genome of Omei Treefrog (*Rhacophorus omeimontis*)

**DOI:** 10.1080/23802359.2019.1698334

**Published:** 2019-12-13

**Authors:** Changkun Fu, Qian Wang, Tao Hu, Ziyong Lei, Hailin Fan, Tianmeng Zhao, Hao Zong

**Affiliations:** College of Life Sciences, Sichuan Normal University, Chengdu, China

**Keywords:** Mitochondrial genome, *Rhacophorus omeimontis*, phylogenetic tree, Omei treefrog

## Abstract

In this study, the complete mitochondrial genome of *Rhacophorus omeimontis* was obtained and described. The sequenced mitogenome is total 19,604 base pairs (bp) in length, which contained 13 protein-coding genes (PCG_S_), 22 transfer RNA genes (tRNA), 2 ribosomal RNA genes (rRNA), and 2 control regions (D-loop). The overall base composition of the mitochondrial DNA is 32.5% for A, 30.5% for T, 23.3% for C, and 13.7% for G, and the percentage of GC content is 37.0%. The complete mitochondrial genome information of *R. omeimontis* will contribute to revealing the phylogenetic relationships among species of family Rhacophoridae.

Omei Treefrog (*Rhacophorus omeimontis*), an arboreal breeder (Liao and Lu 2011), belongs to Rhacophoridae family (Zhao and Adler 1993), is endemic to mountain range in subtropical forests in western China, where it persists at altitudes ranging from 700 to 2000 m above sea level, and its type locality is Mount Emei (Fei and Ye 2001). The genetic variation of Omei treefrog was driven by geologic events and pleistocene climatic oscillations (Li et al. 2015). Some researchers think that Omei treefrog belongs to the genus *Rhacophorus* (Fei et al. 2012), while others think that Omei treefrog belongs to the genus *Zhangixalus* (AmphibiaChina, 2019). In this study, we sequenced complete mitochondrial genome of *R. omeimontis* and reconstructed phylogenetic tree with other 16 species to infer their taxonomic status.

The specimen was collected from Mount Emei (Latitude: 29°34′20.01″N, Longitude: 103°23′41.63″E, Altitude: 751 m), and stored in the Zoological Museum (Specimen number: EM1906001), College of Life Sciences, Sichuan Normal University, China. The complete mitogenome was obtained by high-throughput sequencing method with Illumina Hiseq 2500 (Tsingke, Tianjin). And the complete sequence of mtDNA genome was submitted to GenBank.

The whole mitochondrial genome of *R. omeimontis* is 19604 bp in length (GenBank accession number: MN427892), which contains 13 protein-coding genes (*ATP6, ATP8, COI, COII, COIII,ND1, ND2, ND3, ND4, ND4L, ND5, ND6*, and *Cytb*), 22 transfer RNA genes (tRNA), 2 ribosomal RNA genes (rRNA) and 2 control regions (D-loop). The base composition is 32.5% for A, 30.5% for T, 23.3% for C, and 13.7% for G. ND6 and eight tRNAs are encoded by the L-strand, whereas all the other genes are encoded by the H-strand. The gene arrangement is consistent with other amphibian genomes (Huang et al. 2019). All PCGS of the mtDNA have a methionine start codon (ATR) except ND2 (ATC) and COI (GTG). The large ribosomal RNA (lrRNA) is 1577 bp in length with an A + T content of 63.1% and the small ribosomal RNA (srRNA) is 926 bp in length with an A + T content of 56.8%.Two control regions located on both sides of ND5 are 1430 bp and 2214 bp in length, respectively.

Based on the concatenated nucleotide sequences of protein-coding genes and 2 rRNAs, the phylogenetic relationships of the *Rhacophorus omeimontis* and the other 16 frogs were constructed by MEGA6.0 using maximum-likelihood (ML) method with 1000 bootstrap replications (Tamura et al. 2013, Huang et al. 2019). The phylogenetic tree (Figure 1) showed that the *Rhacophorus omeimontis* was closer to *Rhacophorus schlegelii* (genus *Rhacophorus*) than *Rhacophorus dennysi* (genus *Zhangixalus*) in genetic relationship. It supports that *Rhacophorus omeimontis* belongs to the genus *Rhacophorus* (Fei et al. 2012). However, the molecular evidence inferred in this study is limited, more mitochondrial genomic information of other tree frogs is necessary in order to elucidate the evolutionary relationships within major lineages of Rhacophoridae.

**Figure 1.**
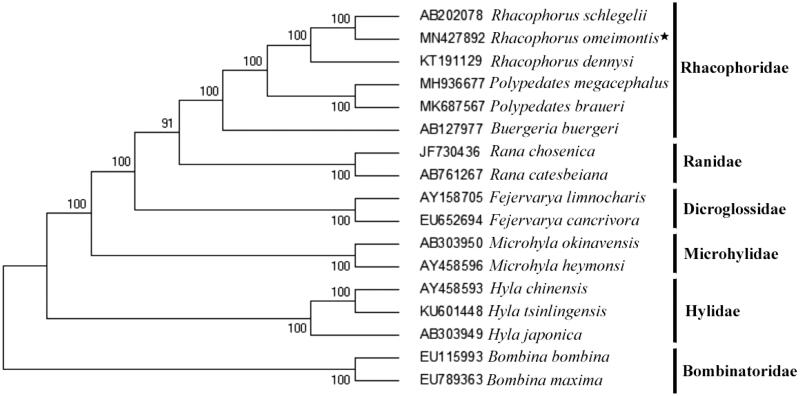
Phylogenetic tree inferred from maximum likelihood analysis of the nucleotide of protein-coding genes and two ribosomal RNA genes. *Bombina bombina* and *Bombina maxima* were used as outgroups. The nodal numbers indicate the bootstrap values obtained with 1000 replicates. The genebank accession number, species name, and family name were shown on the right side of the phylogenetic tree. The newly sequenced mitogenome is indicated by the asterisk.
